# 3D doppler ultrasound imaging of cerebral blood flow for assessment of neonatal hypoxic-ischemic brain injury in mice

**DOI:** 10.1371/journal.pone.0285434

**Published:** 2023-05-09

**Authors:** Guofang Shen, Kayla Sanchez, Shirley Hu, Zhen Zhao, Lubo Zhang, Qingyi Ma

**Affiliations:** 1 Department of Basic Sciences, The Lawrence D. Longo, MD Center for Perinatal Biology, Loma Linda University School of Medicine, Loma Linda, CA, United States of America; 2 Department of Hematologic Malignancies Translational Science, Beckman Research Institute of City of Hope, Duarte, CA, United States of America; 3 Zilkha Neurogenetic Institute and Department of Physiology and Neuroscience, Center for Neurodegeneration and Regeneration, Keck School of Medicine, University of Southern California, Los Angeles, CA, United States of America; Ehime University Graduate School of Medicine, JAPAN

## Abstract

Cerebral blood flow (CBF) acutely reduces in neonatal hypoxic-ischemic encephalopathy (HIE). Clinic studies have reported that severe CBF impairment can predict HIE outcomes in neonates. Herein, the present study uses a non-invasive 3D ultrasound imaging approach to evaluate the changes of CBF after HI insult, and explores the correlation between CBF alterations and HI-induced brain infarct in mouse pups. The neonatal HI brain injury was induced in postnatal day 7 mouse pups using the Rice-Vannucci model. Non-invasive 3D ultrasound imaging was conducted to image CBF changes with multiple frequencies on mouse pups before common carotid artery (CCA) ligation, immediately after ligation, and 0 or 24 hours after HI. Vascularity ratio of the ipsilateral hemisphere was acutely reduced after unilateral ligation of the CCA alone or in combination with hypoxia, and partially restored at 24 hours after HI. Moreover, regression analysis showed that the vascularity ratio of ipsilateral hemisphere was moderately correlated with brain infarct size 24 hours after HI, indicating that CBF reduction contributes to of HI brain injury. To further verify the association between CBF and HI-induced brain injury, a neuropeptide C-type natriuretic peptide (CNP) or PBS was intranasally administrated to the brain of mouse pups one hour after HI insult. Brain infarction, CBF imaging and long-term neurobehavioral tests were conducted. The result showed that intranasal administration of CNP preserved ipsilateral CBF, reduced the infarct size, and improved neurological function after HI brain injury. Our findings suggest that CBF alteration is an indicator for neonatal HI brain injury, and 3D ultrasound imaging is a useful non-invasive approach for assessment of HI brain injury in mouse model.

## Introduction

Hypoxic-ischemic encephalopathy (HIE) is a neurologic condition that results from brain injury in neonates due to perinatal conditions such as pre-eclampsia and birth asphyxia. HIE is estimated to occur in 1–8 per 1000 births and is one of the leading causes of morbidity and mortality in infants worldwide [1,2]. At present, there are very limited therapeutic options available besides hypothermia [3]. Alteration in cerebral blood flow (CBF) and perfusion is the predominant factor contributing to brain damage in the development brain, and may be related to the development of HIE [4,5]. Clinic studies have shown that severe CBF impairment can predict HIE outcomes in neonates [6,7].

The Rice-Vannucci model of neonatal HIE is the most widely used animal model to mimic HI brain injury [8]. It involves ligation of the common carotid artery (CCA) to decrease CBF to the ipsilateral hemisphere followed by hypoxia exposure. However, this model suffers from considerable inter-subject variability in the brain infarct size, partly due to individual differences in collateral compensations and autoregulation capacity in the immature brain [9]. The reasons for the variability in the infarct size have not been precisely identified, which may be attributed to the variability in CBF in the neonatal brain. Approaches including laser speckle have been used to measure CBF alterations in HI brain injury animal models [10–12], and suggest that CBF changes during reperfusion can predict later HI-induced brain damage in mouse or rat pups [10]. Thus, monitoring CBF may provide an option for rapidly and acutely detecting HI brain injury in animal model instead of just brain infarct measurement.

Ultrasound is a non-invasive imaging technique that is easily accessible for monitoring CBF clinically. It has been reported that hemodynamic changes in the middle cerebral artery (MCA) detected by cranial ultrasound can be used for screening of stroke or neonatal HIE [12–14]. However, the use of ultrasound imaging to evaluate CBF changes and analyze whether CBF changes are correlated with the extent of HI brain injury is very limited. Herein, we established a method with 3D ultrasound imaging to monitor CBF, MCA blood flow in a mouse model of neonatal HIE, and examined their relationship with brain infarct. Our results indicate that 3D ultrasound imaging of CBF is an effective method to reflect the severity of HI-induced brain injury in mouse pups, which can be used in the screen of neuroprotectant for neonatal HI brain injury.

## Material and methods

### Animals and surgical procedures

All procedures and protocols were approved by the Institutional Animal Care and Use Committee of Loma Linda University and followed the guidelines by the National Institutes of Health Guide for the Care and Use of Laboratory Animals. Pregnant CD1 mice purchased from Charles River Laboratories (Portage, MI, USA) and their offspring were used for this study. Mice were housed under a 12-h light–dark cycle in Loma Linda University Animal Care Facility and had access to food and water ad libitum. A total of 168 pups of both sexes were used in the present study. Pups without brain lesion after HI insult were excluded. Rigor study criteria were followed including randomization, blinding, predefined exclusion and inclusion criteria, etc. Animals were sacrificed using isoflurane inhalation. Post-operative ketoprofen (2mg/kg, IM) was administered as necessary to relieve any signs of pain.

A modified Rice-Vannucci model was produced in postnatal day 7 (P7) mouse pups modified from the rat model as described previously [8,15–18]. Briefly, mouse pups were fully anesthetized with inhalation of 2–3% isoflurane. The right common carotid artery (CCA) in the neck was exposed, double ligated with an 8.0 silk surgical suture, and then cut between two ligation sites. After surgery, pups were recuperated on a heating pad for 1 h at 37°C, and then placed in a hypoxic incubator containing humidified 8% oxygen balanced with 92% nitrogen for 20 min at 37°C. At the end of hypoxia, pups were returned to their dams for recovery. Mouse pups of mixed males and females were randomly assigned into each experimental group.

### Head ultrasound

Postnatal mouse pups were anesthetized by inhalation of isoflurane (4% for induction, 1.5–2% for maintenance). Mice were placed on the heating pad to maintain core temperature at 37°C. After hair removal, ultrasound transmission gel (Aquasonic 100, Fisher Scientific, Waltham, MA) was then evenly spread on the head to create a conductive interface for the transducer. The MX400 transducer was attached to a motor and lowered to the position. Ultrasound scans were obtained by a small animal ultrasound scanner (Vevo 3100, Fujifilm VisualSonics, Japan) using 3D color doppler mode. Coronal scans were obtained under 2 kHz, 5khz and 8 kHz pulse repetition frequencies (PRFs). The vascularity in the left and right hemisphere of those mice was quantified using volumetric analysis in Vevo LAB 5.5.1. The ratio of the ipsilateral vascularity to contralateral vascularity was calculated to represent CBF impairment in those mice.

### Intranasal drug delivery

The intranasal delivery of CNP was conducted 1 hour after HI insult, as we previously reported [19]. Briefly, pups were placed in the head down-and-forward position. After sedated, droplets of 4 ul saline or saline containing 1000 ng CNP (1–22, #3520, Tocris) were delivered into each naris using a fine tip. The pups were then maintained sedated with isoflurane for 2 min on their backs. All pups woke up within 1–2 min upon withdrawal of isoflurane and were returned to their dams.

### Measurement of brain infarct size

Brain infarct size was determined 24 h after HI using 2, 3, 5-triphenyltetrazolium chloride monohydrate (TTC, Sigma-Aldrich) staining as we previously described [15,18–20]. Briefly, the brain was isolated from each pup, dissected into coronal sections (2 mm thickness, 4 slices per brain), and immersed into pre-warmed 2% TTC in PBS for 5 min at 37°C against light. Sections were washed with PBS, and then fixed by 10% formaldehyde overnight. The caudal and the rostral surfaces of each slice were photographed using a digital camera, and the percentage of infarct area (average of both sides) in the ipsilateral hemisphere for each slice was traced and analyzed by the NIH Image J software.

### Neurobehavioral assay

The sensory motor function of the mouse pups was tested at one month after HI insult by the foot-fault test, which measured the forelimb misplacement on a grid during locomotion. The performance of mouse was videotaped for 6 minutes. The total number of steps and times each forelimb fell below the grid was counted by an observer blinded to experimental groups. The percentage of foot-faults for contralateral forelimb misplacement to total steps was calculated as we reported previously [18]. Animals that did not take enough steps in the 6 minutes were excluded.

### Statistical analysis

All data was plotted and analyzed in GraphPad Prism 8.4 (San Diego, SD, USA). Final data was expressed as mean ± standard error of the mean (SEM). Multiple comparisons were analyzed using one-way ANOVA followed by Newman–Keuls post hoc test. Univariate linear regression of the CBF ratio and infarct size data was conducted in GraphPad Prism. A p value less than 0.05 was considered significant.

## Results

### Acute CBF impairment in the brain of mouse pups after hypoxic-ischemic insult

**Fig 1:**
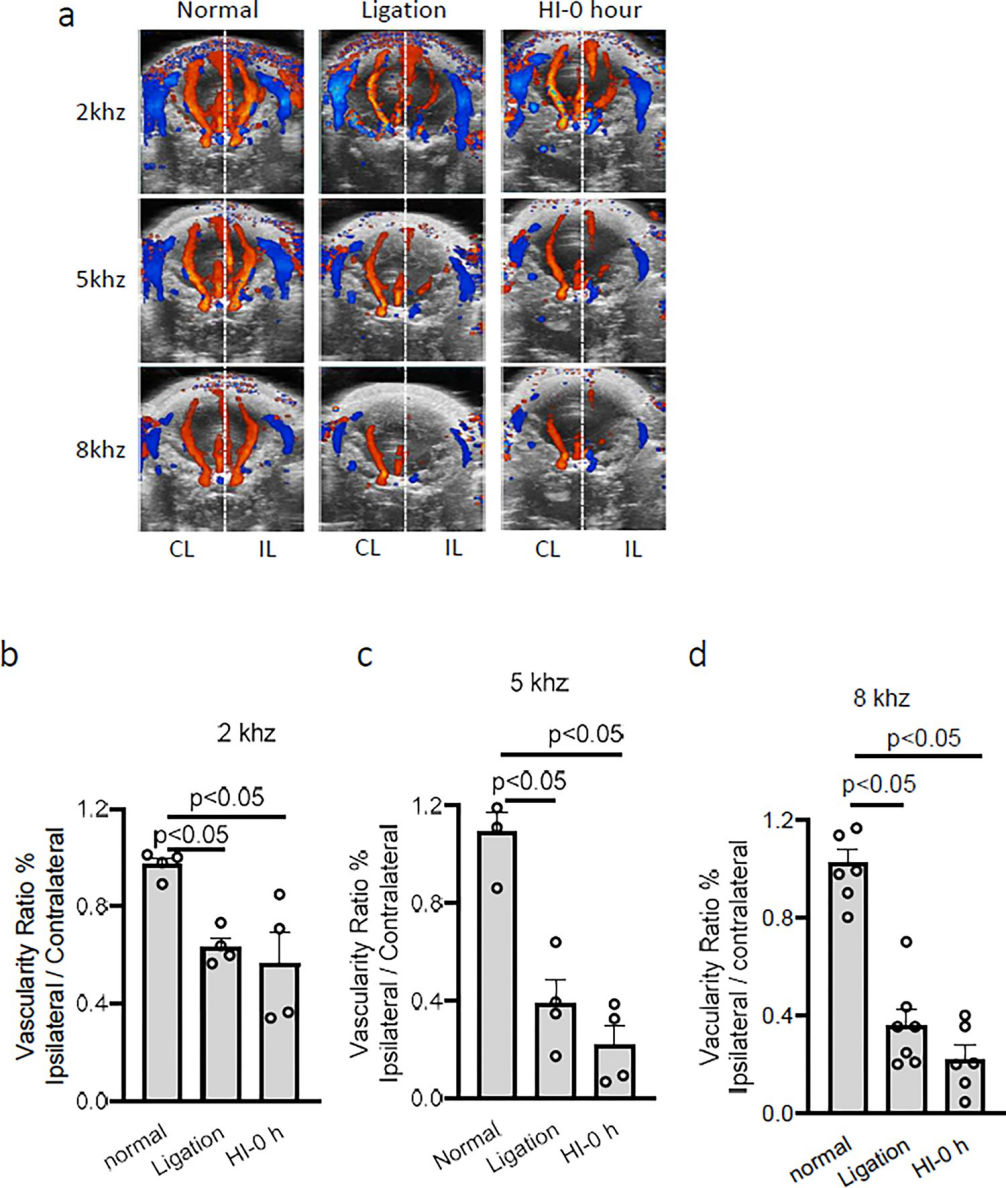
Unilateral ligation of the common carotid artery acutely reduced the ipsilateral cerebral blood flow in the ipsilateral hemisphere of mouse pups. a) representative 2D maximum projection images of the 3D ultrasound scans performed before HI operation (normal), immediately after CCA ligation (ligated), or 0 hour after HI (HI-0 hour) with PRFs 2, 5, 8 kHz. b) The ratio of vascularity in the ipsilateral hemisphere to contralateral hemisphere detected with PRFs of b) 2, c) 5, or d) 8 kHz. b, c, n = 4 pups; d, n = 6–7 pups. CL, Contralateral hemisphere; IL, Ipsilateral hemisphere.

To optimize the parameters for CBF detection, head ultrasound was performed under three chosen pulse repetition frequencies (PRFs) 2, 5 or 8 kHz. A ratio of ipsilateral vascularity to contralateral vascularity (ratio_i/c_) was calculated to reflect CBF impairment. Head ultrasound imaging was performed before CCA ligation (Normal), immediately after ligation (Ligation), and immediately after hypoxia treatment (HI-0 hour). The result showed that the ipsilateral and contralateral hemispheres had similar levels of CBF under different PRFs (0.97±0.03, 2 kHz; 1.09 ±0.08, 5 kHz; 1.07±0.07, 8 kHz) before CCA ligation (Fig 1A–1D, Normal). Immediately after CCA ligation, a reduction of CBF was observed in the ipsilateral hemisphere (Fig 1A, Ligation), which was further reduced after hypoxia treatment detected immediately (Fig 1A, HI-0 hour). In addition, compared with 2 kHz, PRFs 5 or 8 kHz showed enhanced reduction of CBF in the ipsilateral hemisphere (Fig 1A). Accordingly, ligation alone or combined with hypoxia significantly reduced the ratio_i/c_ under different PRFs, compared with the Normal group (Fig 1B–1D), but there was no significant difference observed between ligation alone and HI-0 hour groups. Moreover, the unilateral ligation of CCA decreased ipsilateral CBF by 34% when signals were detected at 2 kHz (Fig 1B), while a 64% or 65% reduction in ratio _i/c_ was observed at 5 kHz (Fig 1C) or 8 kHz, respectively (Fig 1D), respectively, suggesting that higher PRFs is more sensitive to detect velocity changes.

### CBF alteration was negatively correlated with cerebral infarct size after neonatal HI insult

**Fig 2:**
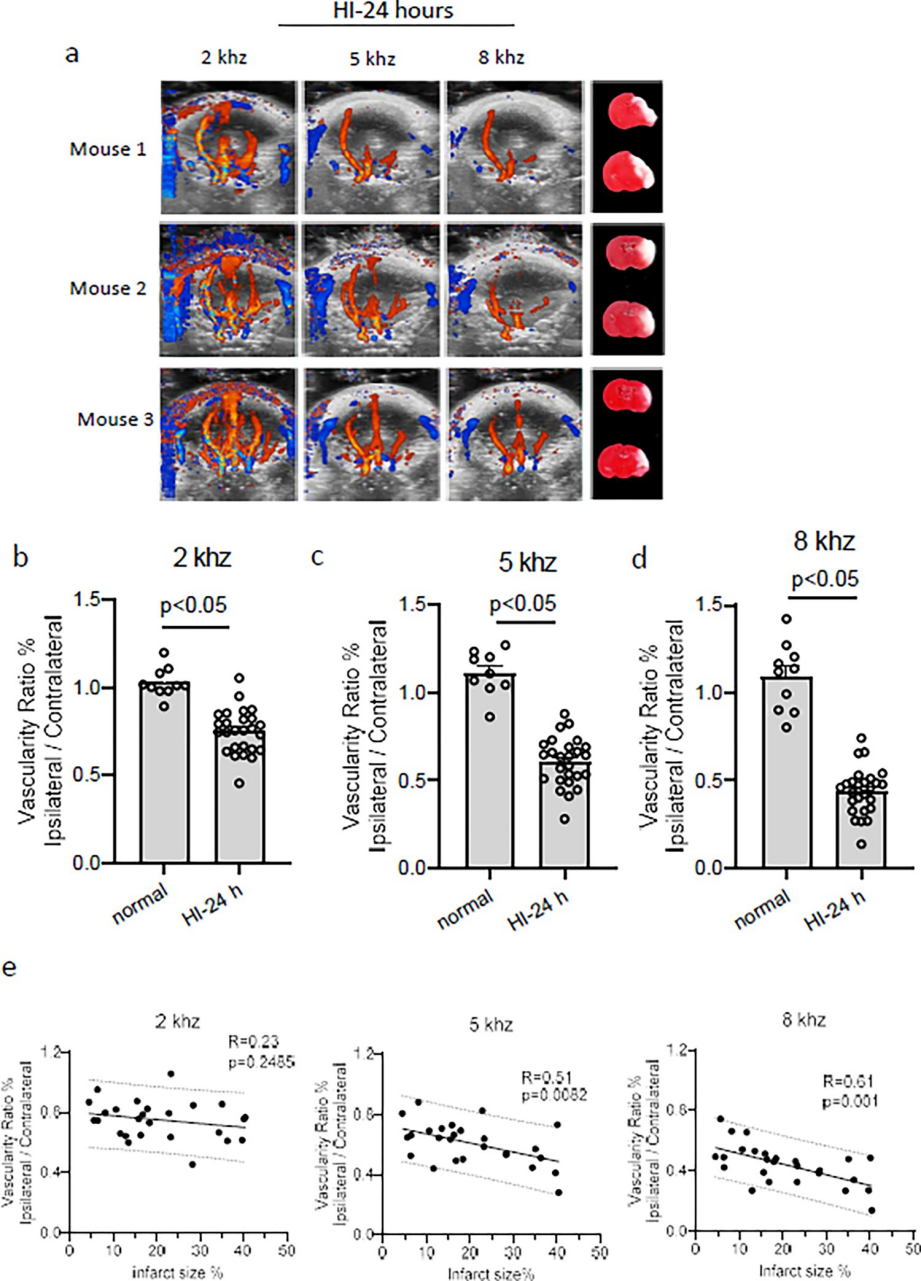
The vascularity ratio is moderately correlated with infarct size 24 hours after HI insult. a) Representative 2D maximum projection images of the 3D ultrasound scans performed on mouse pups 24 hours after HI and images of related brain infarct detected by TTC staining. b-d) The ratio of vascularity in the ipsilateral hemisphere to contralateral hemisphere detected with PRFs of b) 2, c) 5, or d) 8 kHz. Normal, n = 10 pups/group; HI-24 h, n = 26 pups/group. e) Regression analysis of the infarct size toward CBF ratio obtained under the PRF of 2, 5, or 8 kHz. n = 26 pups/group.

We next detected the CBF changes 24 hours after HI insult with different PRFs, in which the brain infarction was developed in HI mouse model and used as an indicator of HI brain injury. The ultrasound result showed that vascularity ratio maintained the significant reduction at 24 hours after HI insult, compared with Normal group (Fig 2B–2D), but increased compared with 0 hour (Fig 1B–1D) after HI operation with PRFs 5 or 8 (5 kHz, HI-0 hour, 0.2207 ± 0.081, vs 24 h, 0.6068 ±0.027; 8 kHz, HI-0 hour 0.2235±0.055, vs 24 h, 0.4396 ± 0.026). We next examined the correlation between alterations of CBF and brain infarct size 24 hours after HI insult. The results showed that pups with smaller infarct size had less impairment in CBF in the ipsilateral hemisphere (Fig 2A). Regression analysis showed a significant moderate negative correlation between the vascularity ratio_i/c_ and cerebral infarct size 24 hours after HI detected with 5 (p = 0.0082) or 8 (p = 0.001) kHz (Fig 2E). This result suggests that the reduction of CBF reflects HI-induced brain infarct in mouse pups, and non-invasive ultrasound is a useful tool for evaluation of HI brain injury.

### Effect of intranasal c-type natriuretic peptide on CBF impairment

**Fig 3:**
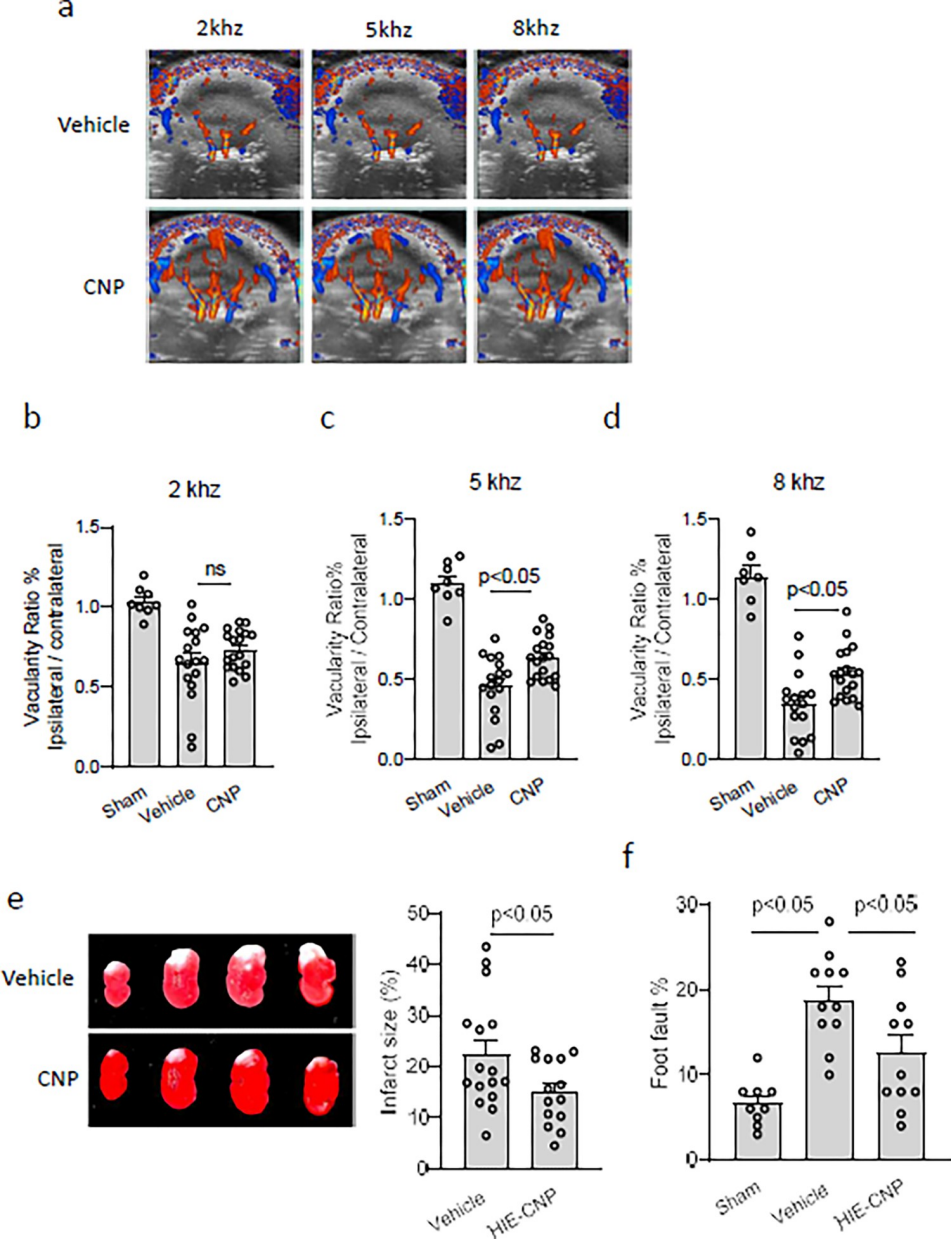
Intranasal CNP improved ipsilateral CBF and protected neonatal brain from hypoxic ischemic brain injury. a) Representative 2D maximum projection images of the 3D ultrasound scans performed on mouse pups with intranasal administration of Vehicle, or CNP. b) The ratio of vascularity between the ipsilateral hemisphere and contralateral hemisphere detected with PRFs of b) 2, c) 5, or d) 8 kHz. n = 8 pups in Sham, n = 17 pups in Vehicle or CNP. e) Representative images of TTC staining and quantitative data of brain infarct size 24 h after HI. n = 14–16. f) Results from foot-fault test performed on mouse pups one month after HI brain injury. n = 9 pups in sham, n = 11 pups in Vehicle or CNP.

Next, we used this non-invasive ultrasound approach to assess the protective effect of neuropeptide c-type natriuretic peptide (CNP) after HI brain injury. One hour after HI insult, either CNP or Vehicle was delivered to the mouse brain through intranasal administration. Ultrasound imaging was performed 24 hours after HI insult. The result showed that CNP treatment significantly reduced CBF reduction, compared with Vehicle under 5 or 8 kHz (Fig 3C and 3D), but no 2 kHz (Fig 3B). TTC staining showed that intranasal CNP treatment significantly decreased brain infarct size 24 hours after HI (Fig 3E), and improved the performance of mouse pups in the foot fault test (Fig 3F) one month after HI.

## Discussion

In the present study, we attempted to use non-invasive color doppler ultrasound to evaluate the brain injury in a neonatal HI mouse model. We demonstrated that the CBF was acutely reduced in the ipsilateral hemisphere of the mouse brain after HI insult, which was somehow recovered, but remains significantly reduced 24 hours after HI insult. Moreover, we identified that CBF alteration was moderately correlated with the brain infarct size induced by neonatal HI insult. We further assessed the neuroprotective effect of CNP administered via intranasal route, and found that intranasal CNP administration protected ipsilateral CBF, reduced brain infarct size, and improved long-term neurological function after HI insult. Our finding suggests that 3D ultrasound imaging of CBF was a useful tool which can be used for evaluation of neonatal HI brain injury in mouse model.

Cranial ultrasound has been widely used in pre-term infants from the 1980s to detect hemorrhage associated with neonatal encephalopathy [21]. Currently, ultrasound still provides a good alternative where MRI is not feasible. As a non-invasive imaging technique, ultrasound provides morphological as well as hemodynamic information of the imaged region [21]. For cranial scans, 7.5 to 10 MHz are adequate frequencies for whole brain scan, and higher frequencies (14–18 MHz) may be used for superficial structures [22]. In animal studies, as the size and depth of the target region differ greatly with that of the human, transducer frequencies ranging from 12–62.5 MHz have been used in different studies [12,23,24]. In the present study, ultrasound scans were obtained at 24 MHz frequency, and cerebral blood flows were successfully detected at this frequency. We used 3D ultrasound scan in the present study to quantify CBF impairment after HI brain injury using volumetric analysis (vascularity%). Other parameters that are thought to be indicative of cerebral tissue injury include hemodynamic parameters such resistance index [6,25], MCA blood flow velocity [12] and cerebral perfusion [23] obtained from 2D scans. The vascularity obtained from the 3D scan has been previously confirmed to be correlated with CBF in mouse model of brain injury [24]. Together, our results support the feasibility of the 3D ultrasound method in monitoring CBF changes in mice.

Compared to 2D scans which generate the flat images, 3D ultrasound scans obtain 3-dimensional images, thus allow volumetric assessment of cerebral blood flows and a more complete evaluation of relative perfusion and blood volume in the region of interest. 3D ultrasound imaging can lay out vessel networks and flow patterns in a complex manner with high-resolution, and therefore may provide additional visual information of target organs, allow for measurement of more parameters such as area, height, length, and volumetric flow of the target anatomy. Many approaches and tools have been used to detect cerebral blood flow changes in neonatal or adult brains in preclinical studies, such as CT scanning, blood flow trackers, color-doppler ultrasound imaging, laser speckling imaging, etc. [26]. Among all these tools, color-doppler ultrasound imaging is most suitable for measurement of hemodynamic parameters of the neonatal mouse brain because it’s non-invasive and has a very short operation time which can reduce the additional stress to mouse pups.

Color doppler ultrasound uses signals that arise from detected doppler shifts to encode the direction and velocity of flows with color. Doppler signal is influenced by multiple physical factors and technical parameters such as Doppler frequency, Pulse repetition frequency and wall filters. The pulse repetition frequency (PRF) is the sampling frequencies of the transducer and adjusting PRF results in simultaneous adjustment of the wall filters (linked control) [27] that may affect machine’s sensitivity to different speed of flow [27,28]. With high PRF settings, lower velocity signals are filtered out by the high-pass wall filter resulting in loss of sensitivity to slow flows [27]. In this study, we used 3 levels of PRF to optimize the detection of CBF in HIE mice. A starting PRF of 2 kHz was chosen to balance inclusion of signals from slow flows and exclusion of motion artifact caused by breathing. We also acquired color doppler scans at 5 or 8 kHz to further filter out motion artifact at the cost of sensitivity to slow flows. Our results showed that 2 kHz PRF can well visualize flows in MCA and filter out most of the motion artifacts. Increased PRF up to 8 kHz decreased the detectable flow signal in the MCA, but blood flow in the contralateral MCA was still well preserved. Our result suggests that 3D color doppler ultrasound with optimized settings is a useful tool to monitor CBF changes especially MCA perfusion in preclinical studies of HIE.

In the mouse brain, left and right internal carotid arteries unite with the circle of Willis that enables interconnection between both hemispheres [29]. Laser speckle imaging studies showed that unilateral ligation of CCA decreased blood flow to ipsilateral MCA despite of compensatory flow through the circle of Willis [10,11]. Hypoxia exposure further decreased CBF on the ipsilateral side and was partially recovered after resuscitation [11]. In line with these findings, our results showed that unilateral ligation of CCA or combined with hypoxia acutely decreased CBF in the ipsilateral hemisphere. We also observed a partial recovery of CBF upon termination of hypoxia exposure 24 hours after HI, suggesting an increased cerebral vascular damage or neuroinflammation [30–32], which may damage the compensation of blood flow to the injury site. Furthermore, we found that the CBF impairment in the ipsilateral hemisphere is moderately corelated with brain infarct size, suggesting that the 3D ultrasound method is possibly used as a non-invasive approach for the assessment of brain injury in HIE model. It is common to see that mice with impaired CBF do not show obvious cerebral infarction. Other factors such as collateral flows may contribute to the resilience of neurons to impaired MCA perfusion. Due to technical limitation, the current ultrasound method is not able to detect blood flows in smaller vessels that may play a role in brain injury development.

CNP is an endothelium-derived peptide that acts as a paracrine factor, causing local vasodilation and preventing smooth muscle cell proliferation [33]. CNP is present in the brain tissues and cerebral spinal fluid and has been reported to exhibit various regulatory functions. Many potential mechanisms underpin the effect of CNP on cerebral blood flow (CBF) restoration after HI brain injury. We have previously demonstrated that intracerebral ventricular delivery of CNP protects neonatal brain from HI-induced neuronal death, endothelial cell injury and BBB disruption [20,34], which promotes cerebral contralateral recruitment and partially restore the CBF in ipsilateral hemisphere after insult [26]. It has been reported that CNP is an important vasodilator which can regulate vascular smooth muscle tone and blood flow [33,35,36]. In addition, previous study showed that CNP inhibits endothelial-leukocyte interactions and platelet reactivity through repression of P-selectin expression in an analogous fashion to NO [37]. Intranasal route of administration is an alternative way for non-invasive brain delivery bypassing the blood brain barrier [38]. The transfer of drug is mediated via the olfactory and trigeminal nerves that connect the nasal passage to the brain, the cerebrospinal fluid, and the lymphatic systems [39]. We use CNP to verify whether 3D ultrasound imaging of CBF can be used as marker for neuroprotectant screen. We found that intranasal CNP improved ipsilateral CBF, reduced infarct size and neurobehavioral deficits in these pups. Thus, the CBF ratio obtained from 3D ultrasound may be used as a non-invasive indicator of physiological impairment in the brain. Future studies are needed to further evaluated the long-term effect of intranasal CNP on CBF changes and the brain injury.

## Conclusion

In conclusion, our study shows that 3D ultrasound is an effective non-invasive method for monitoring acute CBF changes and brain injury in mouse pups after HIE. Using this approach, we demonstrate that intranasal CNP improves brain perfusion and protects the neonatal brain from HI brain injury.
